# Developing a phenotype risk score for tic disorders in a large, clinical biobank

**DOI:** 10.1038/s41398-024-03011-w

**Published:** 2024-07-28

**Authors:** Tyne W. Miller-Fleming, Annmarie Allos, Emily Gantz, Dongmei Yu, David A. Isaacs, Carol A. Mathews, Jeremiah M. Scharf, Lea K. Davis

**Affiliations:** 1 Vanderbilt Genetics Institute, Vanderbilt University Medical Center, TN Nashville, USA; 2 Division of Genetic Medicine, Department of Medicine, Vanderbilt University Medical Center, Nashville, TN USA; 3 Department of Cognitive Science, Dartmouth College, Hanover, NH USA; 4 Department of Pediatric Neurology, Children’s Hospital of Alabama, Birmingham, AL USA; 5 Department of Neurology, Vanderbilt University Medical Center, Nashville, TN USA; 6 Department of Pediatrics, Monroe Carell Jr. Children’s Hospital at Vanderbilt, Nashville, TN USA; 7 Psychiatric and Neurodevelopmental Genetics Unit, Center for Genomic Medicine, Massachusetts General Hospital, Boston, MA USA; 8 Stanley Center for Psychiatric Research, Broad Institute of MIT and Harvard, Cambridge, MA USA; 9 Department of Psychiatry, Genetics Institute, Center for OCD, Anxiety and Related Disorders, University of Florida, Gainesville, FL USA; 10 Department of Biomedical Informatics, Vanderbilt University Medical Center, TN Nashville, USA; 11 Department of Psychiatry and Behavioral Sciences, Vanderbilt University Medical Center, TN Nashville, USA; 12 Department of Molecular Physiology and Biophysics, Vanderbilt University, TN Nashville, USA

**Keywords:** Psychiatric disorders, Psychology

## Abstract

Tics are a common feature of early-onset neurodevelopmental disorders, characterized by involuntary and repetitive movements or sounds. Despite affecting up to 2% of children and having a genetic contribution, the underlying causes remain poorly understood. In this study, we leverage dense phenotype information to identify features (i.e., symptoms and comorbid diagnoses) of tic disorders within the context of a clinical biobank. Using de-identified electronic health records (EHRs), we identified individuals with tic disorder diagnosis codes. We performed a phenome-wide association study (PheWAS) to identify the EHR features enriched in tic cases versus controls (*n* = 1406 and 7030; respectively) and found highly comorbid neuropsychiatric phenotypes, including: obsessive-compulsive disorder, attention-deficit/hyperactivity disorder, autism spectrum disorder, and anxiety (*p* < 7.396 × 10^−5^). These features (among others) were then used to generate a phenotype risk score (PheRS) for tic disorder, which was applied across an independent set of 90,051 individuals. A gold standard set of tic disorder cases identified by an EHR algorithm and confirmed by clinician chart review was then used to validate the tic disorder PheRS; the tic disorder PheRS was significantly higher among clinician-validated tic cases versus non-cases (*p* = 4.787 × 10^−151^; *β* = 1.68; SE = 0.06). Our findings provide support for the use of large-scale medical databases to better understand phenotypically complex and underdiagnosed conditions, such as tic disorders.

## Introduction

Tic disorders (TD) are the most common movement disorder in children and are characterized by sudden and recurrent movements and/or vocalizations [1–4]. While many tic symptoms resolve within a year, persistent TD can cause disruptions to daily life and may have long-term effects on an individual’s social, physical, and mental health [5–7]. TD is highly comorbid with several other psychiatric and neurodevelopmental conditions, including obsessive-compulsive disorder (OCD), attention-deficit/hyperactivity disorder (ADHD), and autism spectrum disorder (ASD), among others [8–17]. One study found that 86% of individuals diagnosed with the most common tic disorder, Tourette syndrome, are diagnosed with at least one additional psychiatric disorder during their lifetime, and up to 58% of Tourette syndrome patients are diagnosed with two or more additional psychiatric disorders during their lifetime [13]. This phenotypic heterogeneity complicates the diagnosis and treatment of patients with Tourette syndrome and other TDs.

Tic disorders are both phenotypically and genetically complex. Heritability measurements for Tourette syndrome range from 0.58 to 0.77 and suggest a strong underlying genetic component; however, identifying the genetic signatures of TD has been difficult [18–20]. Familial and patient studies have identified candidate genes, none of which have been confirmed in independent investigations [21–29]. Genome-wide association studies of TD and Tourette syndrome demonstrate high polygenicity and have identified a few genome-wide significant signals, including Collagen Type XXVII Alpha 1 chain, *COL27A1* and Fms Related Receptor Tyrosine Kinase 3, *FLT-3*, though neither locus has been replicated in an independent study to date [30–32]. This lack of replicable signals is likely due to the polygenic nature of TD, in addition to the challenge of recruiting large cohorts of TD patients [33].

Electronic health records (EHRs) are a useful resource for studying disease outcomes and comorbidities [34]. EHR systems often date back decades and document a wide range of phenotype information, including diagnosis and billing codes, clinician notes, medical histories, lab results, medications, and procedural codes. Additionally, EHR systems allow for the investigation of individuals across diverse disease groups without requiring the resources needed to recruit large cohorts of individuals for genomic studies. In the case of phenotypically complex conditions such as TD, EHRs can provide dense phenotype information spanning before and after diagnosis.

The recent use of phenotype risk scores (PheRS) calculated from medical records has successfully identified patients that exhibit overlapping features of disease. Similar to genetic risk scores, a discovery cohort is used to identify the phenotypic features that characterize a disease or condition in the medical records. These features are then evaluated within an independent target population, and each individual is assigned a score based on the number of features they exhibit. Individuals with a high PheRS are phenotypically similar to the diagnosed individuals of the discovery dataset, whereas individuals with a low PheRS share little or no “phenome” (i.e., set of all phenotypes expressed) with diagnosed individuals. The PheRS method was initially developed to identify patients with features of Mendelian diseases within the medical record database but has recently been applied to common neuropsychiatric conditions, including major depressive disorder, generalized anxiety disorder, and posttraumatic stress disorder [35–38]. This method condenses the medical phenome into a single quantitative score, which can then be used for downstream analyses, making this tool useful for the evaluation of phenotypically complex conditions. Additionally, because this method relies solely on the collection of diagnosis codes within the medical record, it is a powerful tool for indexing liability for TD, given that tics are a common comorbidity but may not be explicitly coded or charted in a medical record. Similar EHR-based machine-learning methods have been successful in the identification of patients with underdiagnosed phenotypes, such as developmental stuttering [39].

In this study we leverage de-identified medical records for 3.6 million individuals from the Vanderbilt EHR system to identify the phenotypic correlates of TD. Using diagnosis billing codes, we identified 1,406 individuals with TD diagnoses and 7,030 age and sex-matched controls. A phenome-wide association study identified 69 phenotypes that were significantly associated with TD diagnosis, including several psychiatric and neurological phenotypes. Using the results of the PheWAS, we generated a PheRS and deployed it within an independent cohort of 90,051 individuals, including 266 individuals with clinician-validated TD diagnoses. We found that the TD PheRS was significantly higher for clinically validated tic patients versus non-diagnosed individuals. This proof-of-concept study: (1) supports the utility of medical records for evaluating the longitudinal effects of phenotypically complex diseases such as TD and (2) provides a framework for using the PheRS as a tool for phenome-wide investigations, improving sample sizes for downstream genetic analyses.

## Results

### PheWAS identified the complex clinical phenome of TD patients

**Fig. 1:**
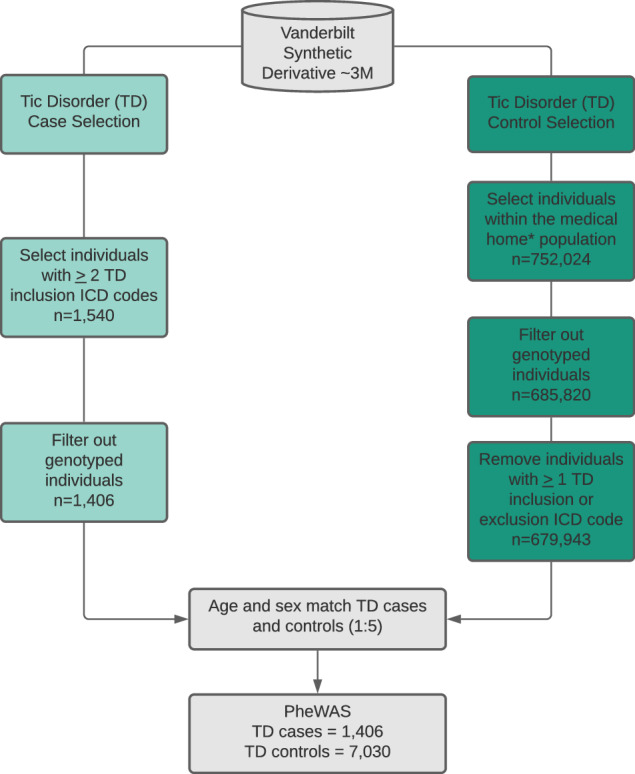
Extraction of tic disorder (TD) cases and controls from electronic health records (EHR). TD cases were identified by selecting individuals with 2 instances of the TD inclusion phenotypes. TD cases were restricted to the non-genotyped population in the EHR. TD controls were matched to TD cases after filtering out the genotyped individuals, those that did not belong to the medical home, and those with at least 1 inclusion or exclusion ICD code for TD. Medical home individuals are those that have visited a Vanderbilt clinic at least 5 times over a three-year period. Matching of cases and controls was performed at a 1:5 ratio, respectively, based on current age and sex. ICD9/10 billing codes used as inclusion/exclusion criteria for TD cases and controls are listed in Supplemental Table 1.

**Fig. 2:**
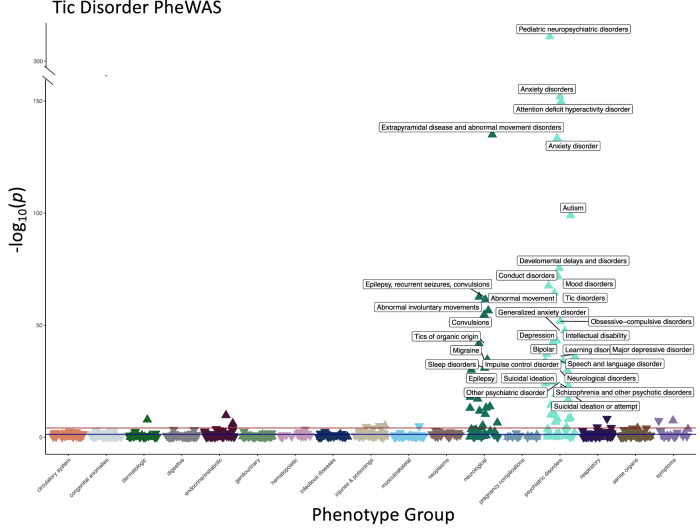
Tic disorder diagnosis PheWAS identifies phenotypic effects across the medical phenome. 676 phenotypes within the electronic health records were tested for enrichment in individuals with ICD9/10 diagnosis codes for TD (*n* = 1406) compared to age and sex-matched controls (*n* = 7030). Phenotypes were defined as phecodes mapped from ICD9/10 billing codes. Logistic regressions were performed for each phenotype, individuals were required to have 2 instances of a phenotype to be considered a case and at least 20 cases were required for testing each phenotype. 69 phenotypes were significantly enriched in the TD cases after Bonferroni-correction (*p* = 7.396 × 10^−5^, 0.05/676 number of tests). Phenotypes with *p* ≤ 5.0 × 10^−20^ are annotated on the Manhattan plot. Covariates included: current age, sex, EHR-reported race, EHR-reported ethnicity, median age of medical record, and number of visits to medical center.

To uncover the phenotypic correlates of TD within a database of de-identified EHR, we performed a PheWAS of TD diagnosis presence/absence (Fig. 1, Supplemental Table 1). As expected for an early-onset disorder with a male-bias, our EHR-derived TD cases (and matched controls) were relatively young (average age 24.22/24.25 years old, average median age of medical record 15.40/13.95 years old, and average age at first ICD code 11.39/9.02 years old in cases/controls) and predominantly male (72.48%/72.59% male in cases/controls). Consistent with the demographic characteristics of the EHR at Vanderbilt, TD cases and controls were significantly skewed for individuals with EHR-reported race and ethnicity as white and non-Hispanic (77.67%/75.02% white in cases/controls and 84.57%/89.20% non-Hispanic in cases/controls) (Table 1). PheWAS analyses stratified by EHR-reported race were difficult to interpret because of the small sample sizes among the non-white populations (Supplemental Table 2), so we performed our PheWAS across all patients and used the EHR-reported race and ethnicity variables as covariates in the PheWAS model. We identified 69 phenotypes significantly associated with TD case status after Bonferroni-correction (*p* < 7.396 × 10^−5^; Fig. 2 and Supplemental Table 2). These phenotypes represent clinical diagnoses, which have been previously mapped from ICD9/10 billing codes extracted from the EHR [40]. Included among the top associations were tic disorders and tics of organic origin, both of which were significantly associated with the TD case label (*p* = 4.18 × 10^−65^; *β* = 9.94; SE = 0.58 and *p* = 1.40 × 10^−42^; *β* = 6.92; SE = 0.51, respectively).

**Table 1:** Demographics for Tic Disorder PheWAS. Tic Disorder (TD) case-control demographics.

	Cases	Controls	*p* value
*N*	1406	7030	–
Sex (%male)	72.48	72.59	0.93
EHR-reported ethnicity	2.63, 84.57, 12.80	7.14, 89.20, 3.66	2.2e-16^b^
(%Hispanic, %Non-Hispanic, %Unknown)			
EHR-reported race^a^	1.3, 6.8, 0.28, 0.14	1.96, 17.4, 0.27,	2.2e-16^b^
(%A, %B, %D, %I, %N, %O, %P, %U, %W)	0.5, 0.07, 0, 13.58, 77.67	0.17, 0.92, 0.03, 0, 4.35, 75.02	
Current age (mean+SD)	24.22 + 14.03	24.25 + 14.01	0.95
Median age of record (mean+SD)	15.40 + 13.30	13.95 + 14.20	2.38e-4^b^
Age at first ICD code (mean+SD)	11.39 + 13.20	9.02 + 13.60	1.18e-09^b^
Age at last ICD code (mean+SD)	18.12 + 13.47	18.06 + 13.89	0.87
Visits (mean+SD)	29.20 + 53.92	25.18 + 37.83	7.86e-3^b^
Medical record length (mean+SD)	6.74 + 5.70	9.04 + 4.43	3.94e-44^b^
ICD code density (mean+SD)	83.21 + 202.04	65.64 + 130.0	0.002^b^
Mean codes per visit (mean+SD)	2.76 + 1.63	2.39 + 1.13	1.50e-15^b^
Median codes per visit (mean+SD)	2.28 + 1.54	1.87 + 0.98	1.59e-20^b^

Tic Disorder (TD) case-control demographics. TD cases (*n* = 1406) were identified using billing codes within the electronic health records. Tic disorder controls (*n* = 7030) were matched to cases based on current age and sex. Independent-samples *t*-test or *χ*^2^ analyses were performed on all measures to determine if there was a statistically significant difference between cases and controls.

^a^EHR-reported race abbreviations: (A): Asian, (B): Black, (D): Declined, (I): Alaskan/Indian, (N): Other Race, (O): Not listed, (P): Pacific Island, (U): Unknown, (W): White.

^b^Denotes statistically significant *p*-value.

### Psychiatric and neurological conditions were highly comorbid in TD patients

As expected, in addition to tic-specific phenotypes, the top signals from the TD PheWAS included known comorbidities, such as: anxiety disorders (*p* = 1.12 × 10^−152^; *β* = 2.38; SE = 0.09) [13], attention-deficit/hyperactivity disorder (*p* = 1.84 × 10^−150^; *β* = 3.43; SE = 0.13) [12], autism (*p* = 1.17 × 10^−99^; *β* = 3.57; SE = 0.17)) [15], developmental delays and disorders (*p* = 3.99 × 10^−76^; *β* = 2.98; SE = 0.16), conduct disorders (*p* = 2.57 × 10^−72^; *β* = 3.41; SE = 0.19) [8], mood disorders (*p* = 2.57 × 10^−68^; *β* = 1.83; SE = 0.10), obsessive-compulsive disorders (*p* = 1.86 × 10^−52^ ; *β* = 4.29; SE = 0.28) [16], depression (*p* = 9.46 × 10^−44^; *β* = 1.73; SE = 0.13) [41], migraine (*p* = 2.70 × 10^−35^; *β* = 1.73; SE = 0.14) [42], and sleep disorders (*p* = 1.19 × 10^−31^; *β* = 1.39; SE = 0.12) [43, 44]. The most significantly associated phenotype was pediatric neuropsychiatric disorders (*p* < 5 × 10^−324^; *β* = 4.80; SE, 0.11), a phecode label that encompasses multiple disorders of impaired communication and socialization skills, including autistic disorders, Asperger’s syndrome, Rett’s disorder, and childhood disintegrative disorder. In the EHR, the phecodes for attention-deficit/hyperactivity disorder (313.1), tic disorders (313.2), and ASD (313.3) are also subcategories within the *pediatric neuropsychiatric disorders* phenotype (313), which explains our finding that 83.36% of the EHR-defined TD cases also had a diagnosis for pediatric neuropsychiatric disorders (Supplemental Table 3).

The TD case label was enriched for several other neuropsychiatric phenotypes, such as bipolar disorder (*p* = 5.11 × 10^−41^; *β* = 2.57; SE = 0.19), suicidal ideation (*p* = 1.38 × 10^−25^; *β* = 2.02; SE = 0.19), schizophrenia and other psychotic disorders (*p* = 1.16 × 10^−24^; *β* = 2.61; SE = 0.25), transient alteration of awareness (*p* = 6.33 × 10^−20^; *β* = 2.16; SE = 0.24), adjustment reaction (*p* = 1.52 × 10^−17^; *β* = 1.46; SE = 0.17), personality disorders (*p* = 1.89 × 10^−13^; *β* = 2.90; SE = 0.39), posttraumatic stress disorder (*p* = 6.93 × 10^−8^; *β* = 1.32; SE = 0.24), and eating disorder (*p* = 2.37 × 10^−5^; *β* = 1.59; SE = 0.38) (Supplemental Table 3). Among EHR-derived TD cases, 85.6% had at least one additional neuropsychiatric disorder diagnosis, and 51.9% had two or more additional neuropsychiatric diagnoses, compared to 21.2% and 16.8%, respectively, within controls. These differences are statistically significant (*p* < 1 × 10^−4^, Fisher’s Exact Test) and are comparable to previous findings of psychiatric comorbidities within individuals with TD [13]. The proportion of control individuals with at least one neuropsychiatric diagnosis was higher than expected (7%), likely because of ascertainment bias of young individuals within the EHR, which tends to be enriched for neurodevelopmental phenotypes. Nevertheless, we found that 47% of the TD cases received an ICD code for psychiatric disorders or neurological conditions at their first medical center visit, compared to 7% of the controls (Supplemental Table 4). Because our EHR database contains longitudinal phenotype information, we examined whether tic diagnoses were more commonly made prior to or after other psychiatric diagnoses. Greater than 64% of the TD cases were diagnosed with a tic disorder before receiving any additional neuropsychiatric diagnoses (Supplemental Table 5). These findings are consistent with the early onset nature of tic disorders and may provide clinicians with critical information regarding the comorbidities that TD patients are at greatest risk of developing later in life.

The neurological phenotypes enriched in TD cases (extrapyramidal disease and abnormal movement disorders (*p* = 1.11 × 10^−135^; *β* = 5.10; SE = 0.21), abnormal movement (*p* = 4.58 × 10^−62^; *β* = 2.00; SE = 0.12), and torsion dystonia (*p* = 6.82 × 10^−20^; *β* = 3.43; SE = 0.38)) may represent comorbidity, misdiagnoses, or the evolution of diagnoses during a diagnostic odyssey. Abnormal movements can also occur in tandem with TD, as a secondary response to TD medication, or independently of TD [45]. To further investigate this, we performed a sensitivity analysis, conditioning the TD PheWAS on the presence or absence of commonly prescribed medications for tics. Associations between hyperkinetic movement disorders and tic disorder diagnosis status remained after correcting for medications, suggesting that these movement phenotype associations were not solely side effects of TD medication usage (Supplemental Table 6**)** [46, 47]. We found that within TD cases, 54% of individuals with a hyperkinetic movement diagnosis received a tic disorder diagnosis first, while the remaining 46% received a movement diagnosis first; however, it is difficult to disentangle these findings, which cannot rule out multiple possibilities for the co-occurring codes, including clinical misdiagnoses (Supplemental Table 7) [45].

**Fig. 3:**
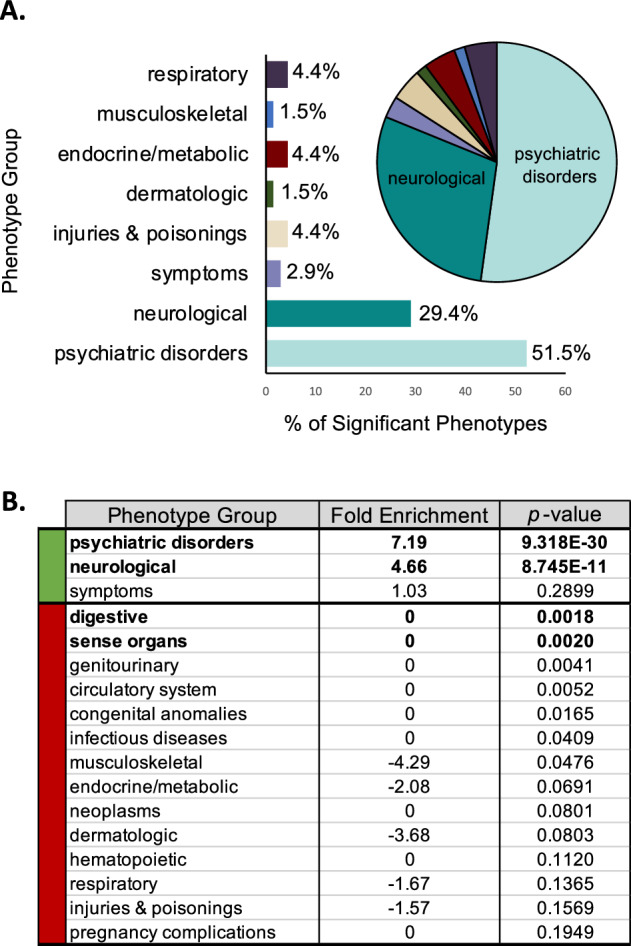
Tic disorder PheWAS is enriched for psychiatric disorders and neurological phenotypes. **A** 69 phenotypes were significantly enriched or depleted within the tic disorder cases versus controls. The bar graph and pie chart show the proportions of these 69 phenotypes across 17 phenotype groups. Over 80% of the phenotypes enriched in the tic disorder individuals belong to the psychiatric disorders or neurological phenotype groups. **B** The phenotype groups for psychiatric disorders and neurological phenotypes are significantly enriched in the tic disorder individuals (7.19- and 4.66-fold enrichment, respectively, *p* < 0.00294 calculated by the hypergeometric test). Digestive disorders and phenotypes within the sense organs were significantly depleted within the TD cases compared to controls (*p* < 0.00294 calculated by the hypergeometric test). Descriptions of the phecode/ICD codes that map to each category have been previously described and can be found here (https://phewascatalog.org/).

The 676 phenotypes assessed in our TD PheWAS were binned across 17 distinct phenotype groups, such as psychiatric disorders, neurological phenotypes, neoplasms, etc. To determine whether a specific phenotype group was over-represented among the 69 significant results, we used a hypergeometric test. Over 80% of the phenotypes enriched in TD cases mapped to psychiatric and neurological disorders (7.19-fold enrichment, *p* = 9.318 × 10^−30^ and 4.66-fold enrichment, *p* = 8.745 × 10^−11^, respectively) (Fig. 3A, B). The remaining significantly enriched phenotype groups included respiratory (4.4%), endocrine/metabolic (4.4%), injuries and poisonings (4.4%), symptoms (2.9%), dermatologic (1.5%), and musculoskeletal (1.5%). These groupings included significant associations with diagnoses such as delayed milestones (*p* = 1.63 × 10^−10^; *β* = 1.22; SE = 0.19), lack of normal physiological development (*p* = 6.52 × 10^−7^; *β* = 0.59; SE = 0.12), and disturbance of skin sensation (*p* = × 1.39 × 10^−8^; *β* = 1.44; SE = 0.25) in TD cases. Controls were significantly enriched for common childhood ailments, including acute upper respiratory infections of multiple or unspecified sites (*p* = 4.91 × 10^−9^; *β* = −0.57; SE = 0.10), fever of unknown origin (*p* = 4.22 × 10^−8^; *β* = −0.72; SE = 0.13), fracture in upper limb (*p* = 9.36 × 10^−6^; *β* = −0.68; SE = 0.15), fracture in hand or wrist (*p* = 2.15 × 10^−5^; *β* = −1.47; SE = 0.35), and cough (*p* = 3.21 × 10^−5^; *β* = −0.59; SE = 0.14) (Supplemental Table 2), consistent with the fact that our control population was age-matched to TD cases and consisted of predominantly young individuals attending the health center for common childhood ailments (average age = 24.25 years, Table 1). The age at first ICD code spans prepubertal and peripubertal ages (9–11.5 years of age) for both the TD cases and controls (Table 1). Despite this range, we did not see significant between-group differences for phenotypes relating to menstruation, hormone regulation, or sexual development (Supplemental Table 2). There was a significant depletion of digestive and sense organ phenotypes in our TD group (*p* = 1.8 × 10^−3^ and *p* = 2.0 × 10^−3^, respectively). Overall, our TD diagnosis PheWAS findings are congruent with established clinical features of TD and reveal the complex phenotypic architecture within individuals diagnosed with TD.

### TD PheRS identified individuals with shared phenotypic features of TD patients

**Fig. 4:**
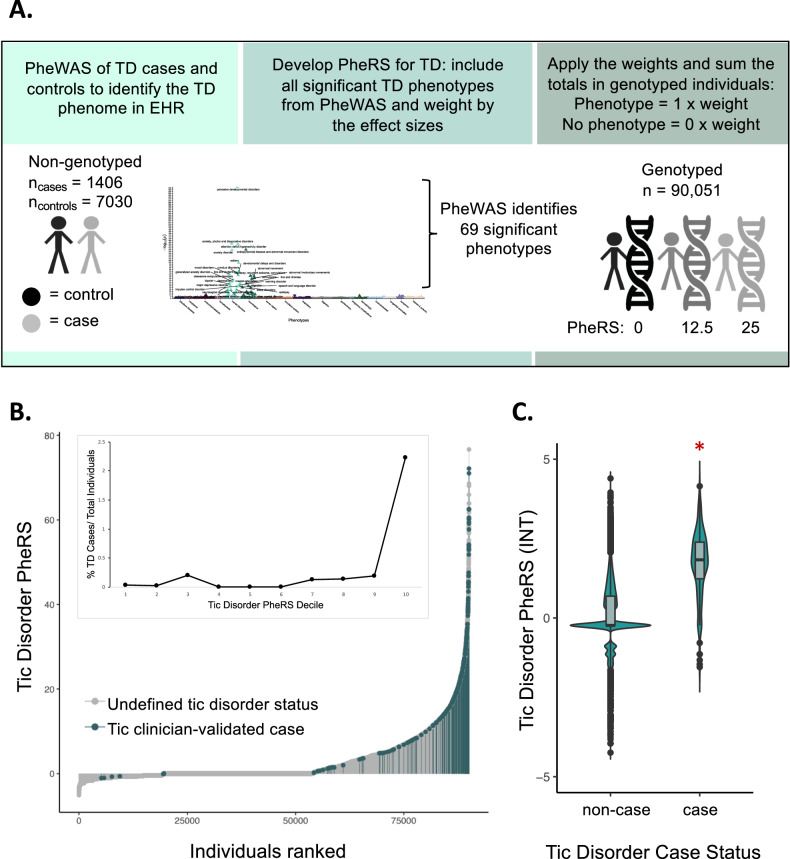
TD Phenotype Risk Score (PheRS) identifies individuals with shared features of tic disorders. **A** The TD PheRS was calculated for 90,051 individuals in the Vanderbilt biobank using the 69 phenotypes identified in the TD PheWAS. Each individual is given a 0 or 1 for the absence or presence of each phenotype and this value is multiplied by the weight of each phenotype (weight = effect estimate from PheWAS analysis). **B** The distribution of the TD PheRS across all genotyped individuals with clinician-validated cases highlighted in blue. All individuals not identified as cases by the TD algorithm and clinician review are gray. Inset shows the percentage of clinician-validated cases within each TD PheRS decile. **C** Violin plots with boxplot insets of the TD PheRS in clinician-validated cases and non-cases shows that the TD PheRS in cases is significantly higher compared to controls (Wilcoxon rank sum test, *p* < 2.2 × 10^−16^, logistic regression analysis *p* = 4.787 × 10^−151^). The TD PheRS was inverse-normal transformed (INT) before plotting. In the logistic regression analysis, which was performed in the individuals of European ancestry, the covariates included PC1-10, current age, median age of medical record, number of medical center visits, and sex.

To consolidate the complex medical phenome of TD into a single, quantitative measure, we constructed a phenotype risk score (PheRS) using the 69 phenotypic features identified by the TD PheWAS (Supplemental Table 2 and Fig. 4A). We identified the PheRS features in the non-genotyped population of the Vanderbilt SD (discovery sample) by performing the PheWAS described above. The significantly associated phenotypes were then used to calculate the PheRS in the genotyped BioVU population (target sample) (Supplemental Tables 8 and 9). As expected, most patients in our target population (59.83%) had a very low TD PheRS because they did not exhibit any phenotypic features of TD (Fig. 4B).

Across the target population, we found that individuals of European ancestry (EA) had a significantly higher mean TD PheRS compared to individuals of African ancestry (AA); however, the EA sample size was much larger which may contribute to this finding (*p* < 2 × 10^−16^; *β* = 0.1; SE = 8.75 × 10^−3^, *n*_EA/AA_ = 70,439/15,174). This finding may also reflect a difference in healthcare utilization between individuals of European and African ancestry, as we found that although these populations had similar medical record lengths, individuals of African ancestry had significantly fewer medical center visits compared to individuals of European ancestry (*p* = 4.72 × 10^−9^; *β* = 5.38 × 10^−4^; SE = 9.17 × 10^−5^). We further found that the TD PheRS was significantly higher in females compared to males across the BioVU population (*p* = 6.02 × 10^−16^; *β* = −0.05; SE = 6.6 × 10^−3^, *n*_female/male_ = 51,183/38,865). Although there is a well-documented male bias among TD, there is literature to suggest that the male bias within TD attenuates with age and that females with TD have more severe and persistent symptoms, consistent with our findings [48–52]. It is also possible that this difference reflects a bias in healthcare utilization, as female patients in BioVU had significantly more clinic visits (*p* = 0.002, *β* = −2.18, SE = 0.69) and significantly longer records (*p* < 2 × 10^−16^; *β* = −1.17, SE = 0.05) compared to male BioVU patients. The TD PheRS was also inversely associated with current age (*p* < 2 × 10^−16^; *β* = −3.91 × 10^−3^; SE = 1.45 × 10^−4^) and median age of medical record (*p* < 2 × 10^−16^; *β* = −3.46 × 10^−3^; SE = 1.46 × 10^−4^), suggesting that younger individuals are more likely to receive codes that contribute to the TD PheRS. Despite being younger, we found that individuals with a higher TD PheRS also had a greater number of visits to the medical center (*p* < 2 × 10^−16^; *β* = 5.38 × 10^−3^; SE = 3.98 × 10^−5^) (Supplemental Table 10). This finding was expected, as the TD PheRS increases with accumulation of relevant phecodes during visits to the medical center.

### TD PheRS was significantly higher in clinically validated tic cases

We previously generated and deployed an algorithm to detect TD cases using information from patient medical records. Cases were identified by the presence of a TD ICD9/10 diagnosis code or a mention of specific tic keywords in their medical records (Supplemental Fig. 1). This approach identified 485 algorithm-defined TD cases, 316 of these were in the target sample. After a clinician chart review of these 316 charts, 266 were confirmed as true TD cases (84.2%).

This subset of clinically validated TD cases in BioVU served as a positive control for the TD PheRS (Supplemental Tables 11 and 12). We found that 75.6% (201/266) of the TD clinician-defined cases had PheRS scores within the tenth decile of the TD PheRS, and 91.4% (243/266) of the cases had PheRS scores falling in the seventh PheRS decile or higher (Fig. 4B). Additionally, we found that clinically validated cases had a significantly higher TD PheRS compared to non-cases according to the Wilcoxon rank-sum test (*p* < 2.2 × 10^−16^) and logistic regression analysis accounting for covariates (*p* = 4.787 × 10^−151^; *β* = 1.68; SE = 0.06; Fig. 4C).

A small proportion of the clinically validated TD cases (23/266, 8.6%) had a very low TD PheRS, falling in the first three deciles (Fig. 4B). These individuals had no ICD9/10 codes in common with TD patients but had mentions of Tourette’s, tics, chronic motor tic disorder, vocal and motor tics, or head tics in their medical records, indicating that these individuals likely visited the medical center for care independent of their tics but included a TD diagnosis within their medical histories. Because the TD PheRS relies solely on the presence or absence of ICD9/10 billing codes and not keywords in the EHR, these individuals had a much lower PheRS despite having a TD diagnosis. Conversely, there were multiple BioVU patients with a high TD PheRS that were not identified by the TD algorithm and clinician validation (825 individuals within the top percentile, Fig. 4B). These individuals share substantial overlapping phenome with TD patients, despite few of them having a TD phecode (15.3%, 126/825). Lastly, within the top percentile of the TD PheRS, over 50% of patients had a broad range of diagnoses including malaise and fatigue, abdominal pain, anxiety disorder, convulsions, nausea and vomiting, depression, major depressive disorder, mood disorders, gastroesophageal reflux disease (GERD), and cough (Supplemental Table 13).

Because several neurodevelopmental disorders are highly comorbid with TD (i.e., ASD, ADHD, and OCD), we tested whether the TD PheRS was able to differentiate between the TD and ASD populations. We used a previously curated set of 444 ASD patients within BioVU. These individuals were identified by ICD9/10 codes and keywords within their charts and were verified by clinician chart review [53]. As expected, we found that the average TD PheRS was highest in the patients with both a TD and ASD diagnosis (mean ± SD = 2.57 ± 0.76) when compared to individuals with only a TD diagnosis (mean ± SD = 2.17 ± 0.72) or only an ASD diagnosis (mean ± SD = 1.85 ± 0.67, Supplemental Fig. 2). We also found that the TD PheRS was significantly higher within the TD patients compared to the ASD patients (*p* = 2.25x10^−6^; *β* = 0.32; SE = 0.07), suggesting that while there was substantial overlap, the TD PheRS was most sensitive to the clinically validated TD individuals (Supplemental Table 10).

## Discussion

The availability of de-identified medical records for research purposes provides an expedited and relatively low-cost way to study phenotypically complex disorders over the lifespan in large patient populations. In this study, we leveraged the large, clinical biobank and EHR database at Vanderbilt to evaluate the phenotypic complexity of TD. We identified TD cases using ICD9/10 diagnosis codes, matched controls, and tested 676 phenotypes within the EHR for enrichment or depletion among TD cases. Consistent with prior studies, we found complex overlapping phenome between TD and several neuropsychiatric phenotypes, with TD most often being the first psychiatric diagnosis in TD cases’ medical records (~64%). Additionally, individuals diagnosed with TD in our sample received an average of 3.1 additional psychiatric diagnoses. Over half of the TD patients (51.9%) received at least two additional psychiatric diagnoses later in life. Comorbid psychiatric disorders often negatively impact patients more than the tics themselves. Psychiatric comorbidities that occurred with increased prevalence in TD cases included anxiety disorders (29.6%), ADHD (19.1%), mood disorders (16.9%), depression (10.7%), ASD (9.4%), and OCD (8.7%) (Supplemental Table 3, Supplemental Table 5). These associations remained significant after conditioning on medication status (Supplemental Table 6). We also found that the associations of TD with lesser-known comorbidities, such as schizophrenia, personality disorders, and transient alteration of awareness, remained after adjusting for medication effects, a finding that warrants future study [54–57]. These findings emphasize the complex phenotypic trajectories for tic patients and support the need for thorough observation and follow-up by clinicians and caregivers even if tic symptoms subside.

Phenotype risk scores can be used in research to quantify the phenomic patterns of comorbidity that characterize TD. This single quantitative score can then be used to investigate broader phenotypic, and even genetic, liability to TD. Indeed, when phenotype prevalence is low, most people with high polygenic risk (i.e., a large number of alleles that are associated with tics) still will not have a TD. However, high genetic liability to TD also increases the odds of related diagnoses (ADHD, OCD, etc.). Thus, a score which quantifies the TD-phenome may provide a path forward to increasing sample sizes for genome-wide association studies of TD liabilities.

In this proof of principle study, we demonstrated that the TD PheRS is significantly higher in confirmed TD cases. Indeed, three quarters of the clinically validated TD cases had a PheRS in the highest decile. Of the 23 confirmed TD cases with a PheRS equal to zero or less, 6 did not meet the medical home criteria despite having mentions of a TD diagnosis in their medical records, making these cases difficult to identify without the use of natural language processing algorithms. For the remaining 17 individuals, despite frequent visits to the medical center and documentation of TD in the clinical notes, the TD phenotype was never coded. Additionally, there were several BioVU individuals in the top percentile of the TD PheRS lacking any TD phecode within their medical record (699/825, 84.7%). These may represent TD patients who are missing a diagnosis code for TD or may represent individuals with several features of TD, such as the several neuropsychiatric comorbidities, without the presence of tics. These findings represent the strength of the PheRS approach at identifying individuals who would have otherwise been labeled as controls and may confound a case-control study because they share many common features with TD patients. The strength of the PheRS lies in the ability to identify individuals with many features of a disease but without a formal diagnosis annotated in the EHR. It is possible that patients with a very high TD PheRS may reach this threshold with co-occurring diagnoses (i.e., ADHD, ASD, OCD) without ever experiencing tics; however, the PheRS approach allows us to identify these patients and review their medical history to develop a more accurate diagnosis. Additional research is needed to determine how informative PheRS phenotypes will be for GWAS of complex phenotypes like TD.

Despite the many conveniences of exploring phenotype information within the EHR, there are substantial limitations. The PheWAS is inherently biased by the number of medical center visits and number of ICD9/10 billing codes collected during these visits. High levels of missing data can exist in a patient’s medical record. A missing diagnosis code does not necessarily reflect the absence of a phenotype and could instead be the result of a patient not receiving all of their care at a single hospital system, or miscoding by the medical center. For these reasons, running case-control studies based on a single diagnosis code can be skewed by misclassification of case and control status. In our approach we have attempted to correct for some of these biases by limiting the controls of our TD PheWAS to the “medical home” population, requiring five diagnosis codes on separate days over three consecutive years, to enrich for individuals that receive the majority of their clinical care at Vanderbilt. Relying on ICD9/10 codes for diagnosis can be risky as institutional and provider coding biases within the EHR exist, especially among phenotypes that have been historically stigmatized, including mental health diagnoses, underscoring the importance of replicating these results in additional EHR systems and validating these approaches with clinician review. The PheRS approach can alleviate some of the EHR coding biases because the score is calculated across several phenotypes to identify patients that share multiple, distinct phenotypic features with the designated case population. Our analyses did not distinguish which Vanderbilt department or clinic individuals from the discovery or target populations visited, which could impact the number and types of codes they received (i.e., specialty neurology clinics versus general healthcare providers). It is also likely that the individuals diagnosed with TD at Vanderbilt represent more severely affected cases due to ascertainment bias of a tertiary care center and this is represented in the proportion of TD cases that received a psychiatric or neurological disorder as their first diagnosis within our medical system (47% of TD cases compared to 7% of controls, Supplemental Table 4). This finding underscores the importance of considering the types of codes being assigned to the case and control populations, as our control samples are enriched for childhood ailments (i.e. fractures, fevers, respiratory symptoms), which thus affects the phenotypes uncovered in the PheWAS which are used in the downstream PheRS construction. Generation of multiple PheRS’s across diverse biobanks should be performed to assess the portability of these methods and examine the effects of different case and control populations.

Ultimately, we find that leveraging the dense phenotype information in a large, clinical biobank can replicate the phenotypic findings of prior studies of TD patients, while adding longitudinal medical outcomes. In addition to the utility of the biobank for research purposes, these tools may also benefit clinicians and TD patients. Our PheWAS reveals multiple comorbid psychiatric phenotypes with TD, which could help clinicians more thoroughly evaluate TD patients and provide necessary interventions and treatments in a timelier manner. The PheRS serves as a quantitative measure encapsulating the broad medical phenome for a given disease. Because the PheRS is diagnosis agnostic and can be applied to target populations with any sample size, this tool could be advantageous for evaluating disease risk based purely on the presentation of phenotypes and provides a phenome-wide perspective when evaluating complex disorders.

## Methods

### The synthetic derivative is a database of de-identified electronic health records

The SD currently houses clinical information and documentation for over 3.6 million individuals who receive clinical care at Vanderbilt University Medical Center (VUMC) dating back to 1994. This information includes insurance billing codes (International Classification of Diseases, 9^th^ and 10^th^ editions/ICD-9 and ICD-10 codes, respectively), clinical procedure codes (Current Procedural Terminology/CPT codes), clinician notes, family histories, lab values, and prescribed medications [58].

### BioVU is a biorepository of genotype data linked to medical records

The Vanderbilt Institute for Clinical and Translational Research at VUMC curates BioVU, a clinical biorepository linked to the de-identified EHR information within the SD [59]. Patients seen at a Vanderbilt clinic are given the option to participate in the BioVU research program, which collects the leftover blood samples from routine clinical testing for genotyping and research. Sample collection for BioVU began in 2007 and is ongoing at Vanderbilt clinics across middle Tennessee. Currently, the BioVU biobank houses DNA samples linked to de-identified EHRs for 329,000 individuals.

### Identification of TD cases and controls in the EHR

We identified TD cases and controls within the EHR using the following criteria. TD cases were required to have at least two separate, temporally distinct instances of case inclusion TD phenotypes, defined by ICD-9 and ICD-10 (International Classification of Diseases, Ninth/Tenth Revision) billing codes (Supplemental Table 1). TD case inclusion codes are: tics (307.2), tic disorder (F95), tic disorder not otherwise specified (307.20), other tic disorders (F95.8), tic disorder unspecified (F95.9), transient tic disorder (307.21/F95.0), tics of organic origin (333.3), other tics of organic origin (G25.69), Tourette’s disorder (307.23/F95.2), and chronic motor or vocal tic disorder (307.22/F95.1). Individuals were excluded from the analysis if any instance of TD exclusion codes (such as non-tic movement disorders) were present in the medical record (Supplemental Table 1). TD cases were restricted to the non-BioVU population within the synthetic derivative (SD) (to allow the BioVU sample to serve as the hold-out validation set), thus yielding 1,406 individuals in the SD who met TD case criteria. Controls were selected from the medical home population within the SD, defined as individuals who had visited a Vanderbilt clinic at least five times within a consecutive three-year period. Controls were restricted to the non-BioVU population within the SD and were age- and sex-matched to the TD cases (5 controls were matched to each case using current age and sex variables) with the MatchIt package in R [60]. Current age reflects the age of the patients at the time of data extraction for this study. Controls were excluded if any instances of either the TD inclusion or exclusion codes were present in the medical record, resulting in 7,030 TD controls. Independent-sample t-tests were performed to test for significant differences in demographic values between TD cases and controls. Chi-squared tests were performed to evaluate differences in proportions of EHR-reported ethnicity and race between cases and controls (Table 1).

### Phenome-wide association study for TD status

A phenome-wide association study (PheWAS) was performed to identify the medical phenotypes enriched in TD cases. TD cases (*n* = 1,406) and matched controls (*n* = 7,030) were assigned a one or a zero case status (exposure variable), and logistic regressions were performed across 676 phenotypes using the PheWAS package in R (versions 0.99.5–2 and 3.6.0, respectively) [61, 62]. The EHR phenotypes (outcome variables), were defined by phecodes mapped from ICD9/10 billing codes across all medical diagnoses as previously described [40]. Individuals were required to have at least two instances of an ICD9/10 code within the medical record to be considered a case for each outcome variable. A minimum of 20 cases was required to test each outcome. Logistic regression model covariates included sex, current age, median age within the medical record, EHR-reported race, EHR-reported ethnicity, and number of visits to the medical center. Phenotypes with an association *p*-value below the Bonferroni-corrected threshold (*p* = 7.396 × 10^−5^, 0.05/676) were reported as significant. In a sensitivity analysis, we conditioned the TD PheWAS on the presence or absence of prescribed medications for tic disorders or common comorbidities (e.g., ASD or ADHD) from the medical records. The medications included in this sensitivity analysis were: amphetamine salts, aripiprazole, atomoxetine, botulinum toxin, bupropion, citalopram, clomipramine, clonidine, clonazepam, desipramine, dexmethylphenidate, dextroamphetamine, duloxetine, escitalopram, fluphenazine, fluoxetine, fluvoxamine, guanfacine, haloperidol, imipramine, lisdexamfetamine, methylphenidate, nortriptyline, olanzapine, paliperidone, paroxetine, pimozide, quetiapine, risperidone, sertraline, tetrabenazine, topiramate, venlafaxine, and ziprasidone [63–65].

### PheWAS phenotype group enrichment

The phecodes analyzed in the TD PheWAS were mapped from ICD9/10 billing codes and were grouped into 17 independent phenotype groups (e.g., psychiatric disorders, neurological conditions, and diseases of the circulatory system). The hypergeometric test was performed to identify phenotype groups that were over- or under-represented in the 69 significant PheWAS results. Phenotype groups with a *p*-value below the Bonferroni-corrected threshold (*p* = 0.00294, 0.05/17) were considered significantly over- or under-represented. The fold enrichment or fold depletion was reported for each phenotype group in Fig. 3B.

### TD phenotype risk score construction

As described above, we performed a PheWAS for TD diagnosis and identified 69 phenotypes significantly enriched or depleted in the TD cases. These 69 phenotypes were then used to construct the TD PheRS in the independent, genotyped BioVU population, or target sample. The following equation was used as previously described:

$${PheRS}_{i}=\mathop{\sum}\limits_{p=1}^{m}{\rm{w}}_{p}\,{x}_{i,p}$$

where *x*_*i,p*_ is equal to 1 if individual *i* has phenotype *p* or 0 if the phenotype is not present in the individual’s health records and *w*_*p*_ refers to the weight of phenotype *p*. The weights for each phenotype are equal to the effect size estimate of each phenotype from the initial PheWAS of the discovery sample. Therefore, phenotypes with the greatest association to TD status contribute most to the PheRS, whereas phenotypes with smaller associations contribute less. Supplemental Table 9 lists the 69 contributing phenotypes with their respective weights. The PheRS was then calculated in 90,051 BioVU individuals (Supplemental Table 8).

### Algorithm and clinician validation of TD cases in BioVU

A combination of TD diagnosis codes and keywords were used to define a TD algorithm that was applied to the BioVU sample. Inclusion criteria required at least two instances of TD ICD-9 billing codes within the EHR (Supplemental Fig. 1) or the single presence of the keywords *motor tic, vocal tic, Tourette*, or *tic disorder* in the clinical notes. Exclusion criteria included ICD-9 billing codes for muscular diseases (Supplemental Fig. 1). The TD algorithm identified 485 cases, 316 of which subsequently underwent clinician chart review because they overlapped with the target BioVU sample. Of the 316 algorithm cases, 266 were clinically validated as true TD cases (84.2%). The patients identified by the TD algorithm that was not confirmed by clinician chart review were excluded as a tic disorder case based on multiple criteria (e.g., having a broad mention of tics in the medical history without additional information, tics that appeared following medication use, and mentions of tic misdiagnosis after appearance of abnormal movements or seizures). Of the 266 clinically validated TD individuals, 95 met the ICD9/10 code criteria for TD case definition, 40 met the keyword criteria, while 131 met both.

### Supplementary information

Supplemental Text and Figures

Supplemental Table 1

## Data Availability

Synthetic derivative (de-identified electronic health records) and BioVU data are available from Vanderbilt University Medical Center with institutional restrictions that govern the acquisition, use, and dissemination of data. Individuals interested in using this data in a non-profit, academic setting can contact the Vanderbilt Institute for Clinical and Translational Research (research.support.services@vumc.org) and request an application to the Integrated Data Access and Services Core.
