# Supplementary material for: Developing a phenotype risk score for tic disorders in a large, clinical biobank
Source: Transl Psychiatry. 2024 Jul 28;14:311. doi: 10.1038/s41398-024-03011-w (PMC11284231; doi:10.1038/s41398-024-03011-w)
Supplement: Supplementary file 1 — Supplemental Text and Figures [file 41398_2024_3011_MOESM1_ESM.pdf]

Developing a Phenotype Risk Score for Tic Disorders in a Large, Clinical Biobank

Tyne W. Miller-Fleming, PhD[1,2], Annmarie Allos, BA[1,3], Emily Gantz, DO[4-6], Dongmei Yu, PhD[7,8], David A. Isaacs, MD, MPH[5,6], Carol A. Mathews, MD[9], Jeremiah M. Scharf, MD, PhD[7,8], Lea K. Davis, PhD[1,2,10-13*]*

Supplemental Figures

A.

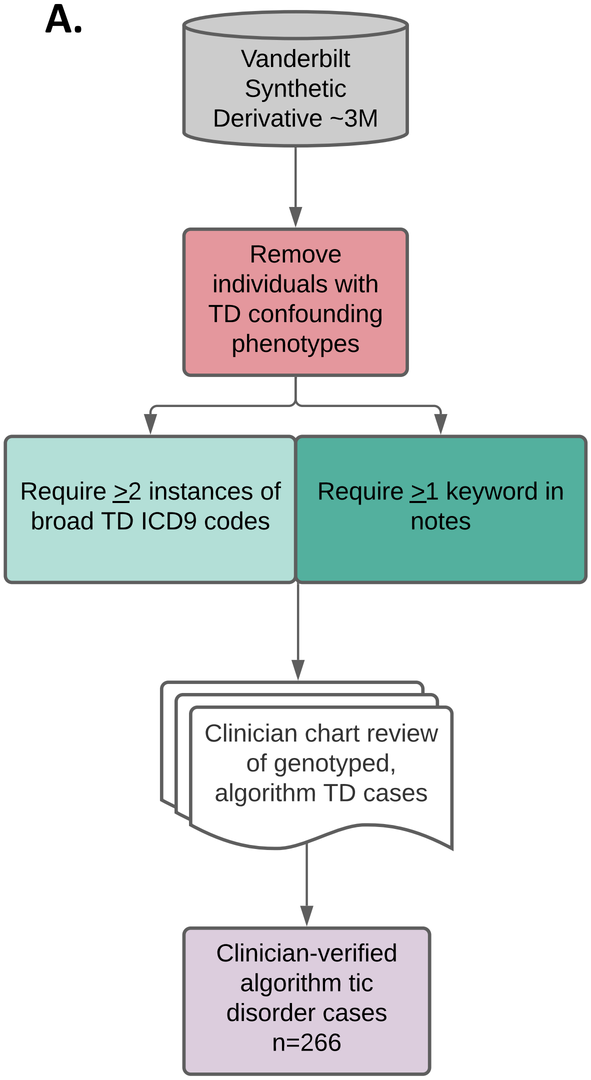

B.

Tic Disorder (TD) Confounding ICD-9 Codes	
ICD-9 Code	Phenotype
333.6	Genetic torsion dystonia
333.7	Acquired torsion dystonia
333.72	Acute dystonia due to drugs
333.79	Other acquired torsion dystonia
333.8	Fragments of torsion dystonia
333.82	Orafacial dyskinesia
333.85	Subacute dyskinesia due to drugs
333.89	Other fragments of torsion dystonia

C.

Broad Tic Disorder (TD) ICD-9 Codes	
ICD-9 Code	Phenotype
307.2	Tics
307.20	Tic disorder NOS
307.21	Transient tic disorder
307.22	Chronic motor or vocal tic disorder
307.23	Tourette's disorder
333.3	Tics of organic origin

D.

Tic Disorder (TD) keywords
motor tic
vocal tic
Tourette
tic disorder

Supplemental Figure 1: Tic Disorder (TD) algorithm and clinician chart review identify tic disorder cases in the EHR.

A. The Synthetic derivative (SD) at Vanderbilt consists of de-identified electronic health records for ~3.6 million individuals. To identify individuals with tic disorders and exclude individuals with confounding phenotypes, patients with torsion dystonia and dyskinesia diagnoses were excluded. The TD algorithm required at least 2 instances of TD ICD-9 codes and 1 TD keyword in the records for each individual. When restricted to genotyped individuals, this algorithm identified 485 TD cases. Clinician chart review was performed on 316 of the 485 individuals which overlapped with

the BioVU target sample, which resulted in 266 clinician-validated TD cases. **B.** Table of TD-confounding phenotypes which were used as exclusion criteria in the algorithm. **C.** Table of TD ICD-9 codes which were used to identify TD cases. **D.** Table of TD keywords which were used to identify TD cases.

A.

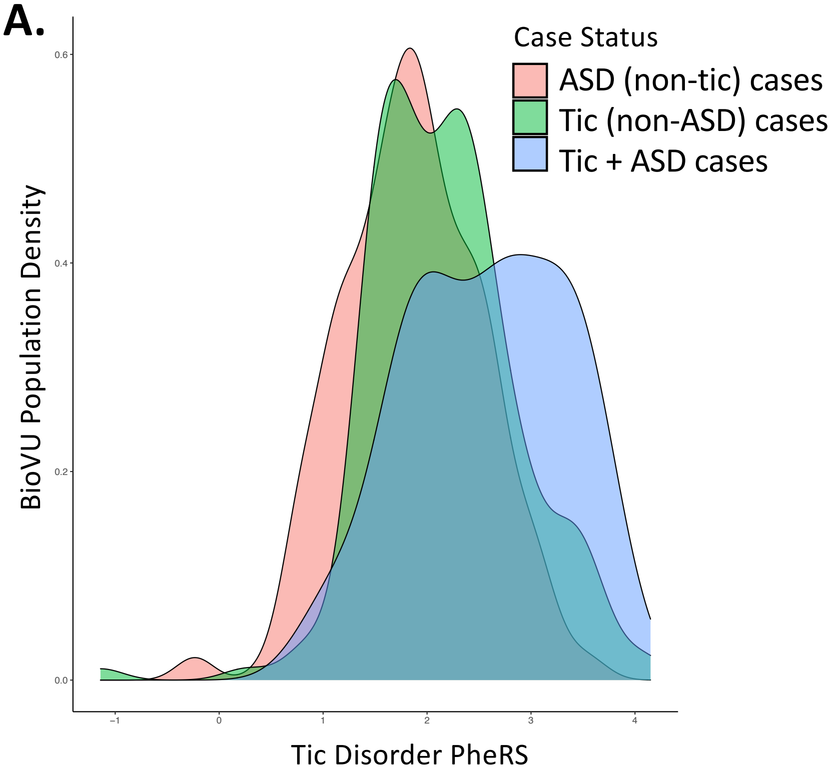

B.

Case Status	N	Mean ± SE TD PheRS
ASD (non-tic)	412	1.85 ± 0.67
Tic (non-ASD)	234	2.17 ± 0.72
Tic + ASD	32	2.57 ± 0.76

Supplemental Figure 2: Performance of the tic disorder PheRS in individuals with autism spectrum disorder (ASD).

We used a previously curated set of clinically validated ASD patients in the EHR, to test whether the tic disorder PheRS can differentiate between tic disorder patients and ASD patients. These individuals were compared to the clinically validated tic disorder patients ascertained from the same BioVU population. **A.** Density plots of the TD PheRS in individuals with ASD only, tic disorders only, or ASD and tic disorders show that patients with a tic diagnosis (non-ASD) have a mean TD PheRS higher than those patients with an ASD diagnosis (non-tic). Additionally,

patients with a diagnosis of both tic disorder and ASD have the highest TD PheRS on average. **B.** Sample sizes and mean TD PheRS values are reported for the three groups: ASD (non-tic) cases, tic (non-ASD) cases, and tic and ASD cases.
